# Supplementary material for: Splice-Junction-Based Mapping of Alternative Isoforms in the Human Proteome
Source: Cell Rep. Author manuscript; Available in PMC 2020 Jan 15. (PMC6961840; doi:10.1016/j.celrep.2019.11.026)
Supplement: 2 [file NIHMS1546469-supplement-2.zip › DF1/PXD009737/TestisTrypsin/TestisTrypsin_71_BAX_TGALLLQGAHQLGGG.pdf]

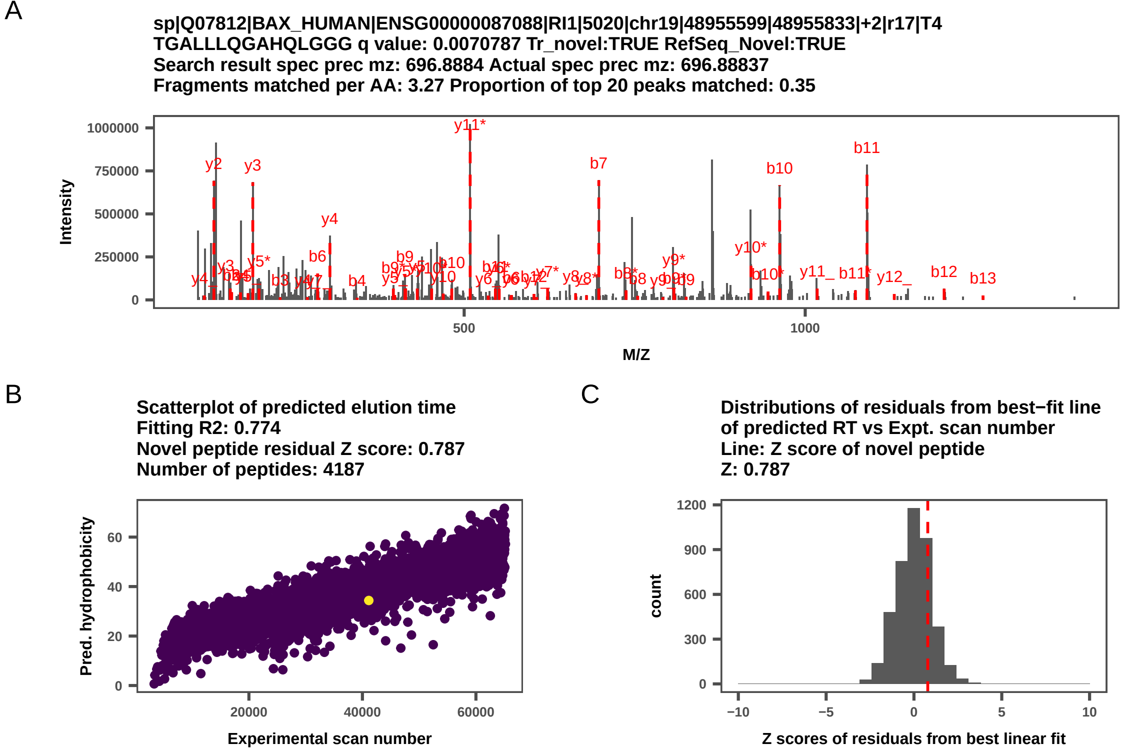
A
sp|Q07812|BAX_HUMAN|ENSG00000087088|RI1|5020|chr19|48955599|48955833|+2|r17|T4
TGALLLQGAHQLGGG q value: 0.0070787 Tr_novel:TRUE RefSeq_Novel:TRUE
Search result spec prec mz: 696.8884 Actual spec prec mz: 696.88837
Fragments matched per AA: 3.27 Proportion of top 20 peaks matched: 0.35
Intensity
1000000
750000
500000
250000
0
y11*
y2
y3
y4
b6
b7
b10
b11
y10*
b12
b13
500
1000
M/Z
B
Scatterplot of predicted elution time
Fitting R2: 0.774
Novel peptide residual Z score: 0.787
Number of peptides: 4187
Pred. hydrophobicity
60
40
20
0
20000
40000
60000
Experimental scan number
C
Distributions of residuals from best−fit line
of predicted RT vs Expt. scan number
Line: Z score of novel peptide
Z: 0.787
count
1200
900
600
300
0
−10
−5
0
5
10
Z scores of residuals from best linear fit
